# Gene expression profile association with poor prognosis in epithelial ovarian cancer patients

**DOI:** 10.1038/s41598-021-84953-9

**Published:** 2021-03-08

**Authors:** Douglas V. N. P. Oliveira, Kira P. Prahm, Ib J. Christensen, Anker Hansen, Claus K. Høgdall, Estrid V. Høgdall

**Affiliations:** 1 Department of Pathology, Herlev Hospital, University of Copenhagen, Herlev, Denmark; 2 Department of Gynaecology, Juliane Marie Centre, Rigshospitalet, University of Copenhagen, Copenhagen, Denmark; 3 Oncology Venture, Horsholm, Denmark

**Keywords:** Biomarkers, Cancer genetics, Cancer genomics, Gynaecological cancer, Tumour biomarkers, Cancer genomics, Gynaecological cancer, Tumour biomarkers

## Abstract

Ovarian cancer (OC) is the eighth most common type of cancer for women worldwide. The current diagnostic and prognostic routine available for OC management either lack specificity or are very costly. Gene expression profiling has shown to be a very effective tool in exploring new molecular markers for patients with OC, although association of such markers with patient survival and clinical outcome is still elusive. Here, we performed gene expression profiling of different subtypes of OC to evaluate its association with patient overall survival (OS) and aggressive forms of the disease. By global mRNA microarray profiling in a total of 196 epithelial OC patients (161 serous, 15 endometrioid, 11 mucinous, and 9 clear cell carcinomas), we found four candidates—*HSPA1A*, *CD99*, *RAB3A* and *POM121L9P*, which associated with OS and poor clinicopathological features. The overexpression of all combined was correlated with shorter OS and progression-free survival (PFS). Furthermore, the combination of at least two markers were further associated with advanced grade, chemotherapy resistance, and progressive disease. These results indicate that a panel comprised of a few predictors that associates with a more aggressive form of OC may be clinically relevant, presenting a better performance than one marker alone.

## Introduction

Ovarian cancer (OC) is the eighth most common cancer for women globally^1^. In 2018, there were approximately 293,000 new cases of OC and 185,000 associated deaths^1^. Due to its asymptomatic characteristics, 65% of all OC cases are diagnosed at an advanced stage (FIGO III-IV), leading to a 5-year overall survival (OS) of patients ranging from 30 to 50%^2^. Cytoreductive surgery followed by adjuvant platinum-based chemotherapy is the current standard treatment of OC patients. The aetiology of this disease is unclear, reflecting the heterogeneity of OC. Nevertheless, defining the disease stage at diagnosis is crucial for predicting prognostic outcome of the patient, such as OS. Currently, CA-125 is used as a biomarker for OC management, such as perioperative assessment for treatment response^3,4^. However, its specificity is very limited, given that CA-125 levels can also associate with menstruation, endometriosis and other inflammatory diseases. Therefore, the discovery and use of new biomarkers are still crucial for better management of OC, such as prediction of OS, progression of the disease, and response to treatment.

Full gene expression profiling has been employed in studies screening for potential molecular biomarkers capable to improve tumour classification and staging, predict chemotherapy response and impact on overall patient outcome^5–7^. Since its development in early 2000s, whole gene expression profiling has been an invaluable tool for the investigation of molecular biomarkers in a plethora of types of cancer. In OC, previous studies have shown a distinct gene expression pattern among patients with short and long survival^8,9^ and association with the metastatic form^10^. Furthermore, a small pool of genes was first described to predict patient sensitivity to conventional platinum-based therapy^11^, shortly followed by a more comprehensive study which validated a smaller panel of genes as predictors for chemotherapy response^12^. Nonetheless, the validation of such findings are generally not addressed when independently applied to external cohorts^13,14^. Epithelial OC (EOC), the most frequent form of OC (approximately 90% of the cases), is a heterogenic disease, consisting of four major histologic subtypes—serous, endometrioid, clear cell and mucinous carcinomas^15,16^. Each one of them present their own morphological and molecular differences. Additionally, the serous subtype can be further categorized into high grade and low grade serous^17,18^.

Here, we primarily investigated the association of gene expression profiles across different subtypes of EOC with overall survival (OS) of patients. Moreover, we further examined whether the discovered genes are also associated with a more severe prognosis of EOC, by observing progression of the disease, tumour grade and chemotherapy resistance. To that end, whole gene expression profiles were performed in a cohort of 196 tissue samples from different subtypes of EOC for target screening. We further combined and classified the candidate markers into two different groups in order to established whether their expression is associated with patient OS, and other clinicopathological characteristics associated to poor prognosis. Given the small panel of biomarkers, that could indicate a perspective use in the clinic. Thus, we have further sought to validate its efficiency in an external cohort, and compared its performance with another gene panel signature^19^.

## Results

### Clinical and pathological features of the patients

Primarily, the first 246 included patients with epithelial OC were identified. From those, 50 patients were excluded due to insufficient tumour material for analysis (n = 24) or technical issues (n = 26). A total of 1,967 patients were eligible for inclusion in the study. Histological subtypes were represented by 162 (82.2%) serous carcinomas, 15 (7.6%) endometrioid carcinomas, 11 (5.6%) mucinous carcinomas and 9 (4.6%) clear cell carcinomas. Early stage diagnoses (FIGO I–II) (International Federation of Gynaecology and Obstetrics) accounted for 52 (26.4%) of the cases, whilst 145 (73.6%) were advanced stage (FIGO III–IV). Low-grade tumours were found in 20 (10.2%) patients, and high grade in the remaining 177 (89.8%) subjects. 39 (19.8%) patients were categorized with type I tumour, and 158 (80.2%) with type II tumour^18^. Briefly, in this study type I carcinomas comprise low grade serous, endometrioid, clear cell, and mucinous carcinomas, whilst type II are largely composed of high grade serous carcinoma, following the proposed classification^20^. Patient demographics are shown in Table 1.

**Table 1:** Clinicopathological characteristics of OC patients.

**Status**	
Alive	64 (32.5%)
Death	133 (67.5%)
Median age in years (range)	64 (31–89)
Median OS^1^ in months	48 (95% CI 40–52)
Median PFS in months (% progressed)	20 (72.9%)
**Histology**	
Serous adenocarcinoma	162 (82%)
Mucinous adenocarcinoma	11 (6%)
Endometrioid adenocarcinoma	15 (8%)
Clear Cell adenocarcinoma	9 (5%)
**FIGO stage**^**2**^	
I–II	52 (26.4%)
III–IV	145 (73.6%)
**Histologic grade**	
1	20 (10%)
2	102 (52%)
3	74 (38%)
Unknown	1 (< 1%)
**Type I or II**	
I	39 (19.8%)
II	158 (80.2%)
**Risk group**^**3**^	
Low	178 (90.8%)
High	18 (9.2%)

^1^OS = overall survival.

^2^FIGO = International Federation of Gynaecology and Obstetrics.

^3^Risk group is based on the 4 found candidates.

### Discovery of mRNA expression associated with overall survival

**Figure 1:**
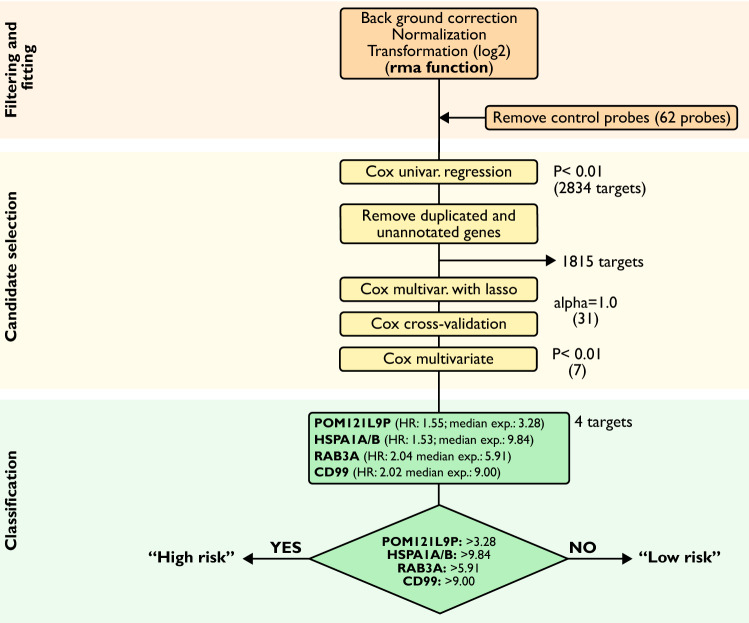
Analysis workflow.

A full profile of gene expression was investigated in order to find association with OS of patients. Given the extensive target panel, we primarily explored association of each gene alone to OS. Thus, univariate Cox regression analysis was performed, followed by annotation and removal of duplicated genes, resulting in 20,175 unique genes for subsequent analysis. A total of 1,019 targets were found as potential predictors (P < 0.01). Furthermore, we evaluated the association of OS with all those targets combined, by applying a multivariate Cox regression model. In order to make a valid model, a lasso-penalty variation was implemented followed by cross validation, resulting in 31 targets. Finally, a multivariate analysis was performed in order to verify those predictors. For a better and robust clinical significance, only candidates with an absolute hazard ratio (HR) above 1.5 were considered. In total, 4 potential targets were identified (HR > 1.5, P < 0.01), *HSPA1A* (HR: 1.53; median expression: 9.84), *CD99* (HR: 2.02; median expression: 9.0), *RAB3A* (HR: 2.04; median expression: 5.91), *POM121L9P* (HR: 1.55; median expression: 3.28) (Table 2). The overexpression of all those targets showed a significant association with poor OS of patients. The analysis workflow is presented on Fig. 1.

**Table 2:** Statistical summary of the 4 discovered targets.

	Log2 expr.^1^	HR	std. error	P
*HSPA1A*	9.842	1.531	0.149	0.0041
*CD99*	8.999	2.016	0.234	0.0027
*RAB3A*	5.907	2.039	0.260	0.0061
*POM121L9P*	3.279	1.549	0.156	0.0051

^1)^Log_2_ gene expression as median values.

### Overexpression of candidate targets predicts poor OS and PFS

**Figure 2:**
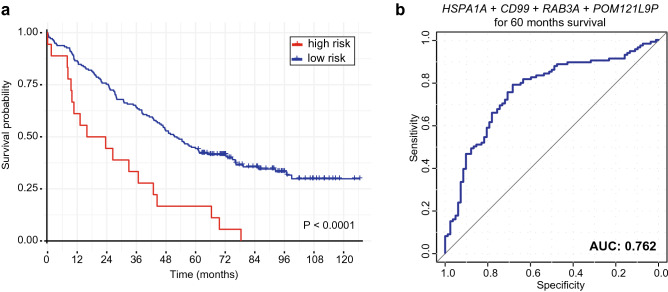
Overall survival and prediction performance of the 4 candidates. (**a**) Survival curve shows that “high risk” (all candidates overexpressed) group has a shorter survival compared to “low risk” (at least one candidate down regulated) OC patients. (**b**) AUC/ROC curve of combination of *HSPA1A*, *CD99*, *RAB3A* and *POM121L9P* for 5-year OS. P value and AUC are presented above.

Based on the fact that the up-regulation of all predictors (*HSPA1A*, *CD99*, *RAB3A*, and *POM121L9P*) showed to be associated with shorter OS, we classified the patients into two different groups: “high risk” and “low risk” for short survival. Patients whose expression level of each gene was overexpressed, value above the median, were classified as “high risk” (Fig. 1). Those that did not fit the criteria were included on the “low risk” group. In order to ensure that our classification was not biased by marginal differences in expression values, we evaluated the overall gene expression profile on both groups. Their levels were significantly different (P < 0.0001) (Supplementary Fig. S1). Moreover, 5-year OS is commonly used in the clinic as a means to observe the efficiency of a treatment, especially in more aggressive diseases, where life expectancy is short. The classification showed distinction between classes, where “high risk” patients had a significantly lower OS in comparison to the “low risk” group, with 5-year OS of 16.7% and 44.4%, respectively (P < 0.0001) (Fig. 2a). Noteworthy, when examined individually, with the exception of *RAB3A*, all targets presented lower indexes performance when compared to all combined (Supplementary Fig. S2). Furthermore, we further investigated the efficiency of those candidates to discriminate between patients with OS longer or shorter than 5 years by calculating the ROC curve, irrespective of their groups. The combination of all markers showed a predictive performance of AUC = 0.76 (Fig. 2b). Because *RAB3A* alone seemed to have presented a similar performance to all 4 targets, we assessed its efficiency in discriminating those patients with longer and shorter survival. Nonetheless, its predictive performance was below that of all targets combined, AUC = 0.69 (Supplementary Fig. S2).

Considering that 82% (161) of samples were derived from serous adenocarcinoma histologic subtype, we investigated whether our observations were due to this factor. We first performed a survival curve analysis in this group alone. The “high risk” and “low risk” groups still showed a distinct outcome, however with a slightly lower gap between them, with a 5-year OS of 16.7% and 37.8%, respectively (P < 0.001) (Supplementary Fig. S3a). Secondly, we compared the survival curves between serous adenocarcinoma with all other subtypes combined to observe their differences. Considering that all “high risk” samples were found only on serous adenocarcinoma, we perform the comparison only in the “low risk” group. There was no difference between sub-groups of EOC (Supplementary Fig. S3b). Furthermore, the combination of *HSPA1A*, *CD99*, *RAB3A*, and *POM121L9P* performed close to baseline in distinguishing serous from all other subtypes (Supplementary Fig. S3c). No difference was detected in gene expression profile among all the histologic subtypes (Supplementary Fig. S4). We have further sought into reclassifying the “high risk” and “low risk” groups based on the median value for each histologic subtype alone. No difference has been observed, further indicated by the similar median expression values (Supplementary Table S1). Finally, considering that high grade and low grade serous adenocarcinomas are morphologically distinct, we also examined the association of the candidates on these groups. The difference between the “high risk” and “low risk” was still observed in the high grade subgroup (Supplementary Figure S5). Given the small sample size of low grade serous, this analysis was not feasible in this group. These results indicate that *HSPA1A*, *CD99*, *RAB3A* and *POM121L9P* may correlate with shorter patient survival mainly in the serous subtype, with indications in the other subgroups.

**Figure 3:**
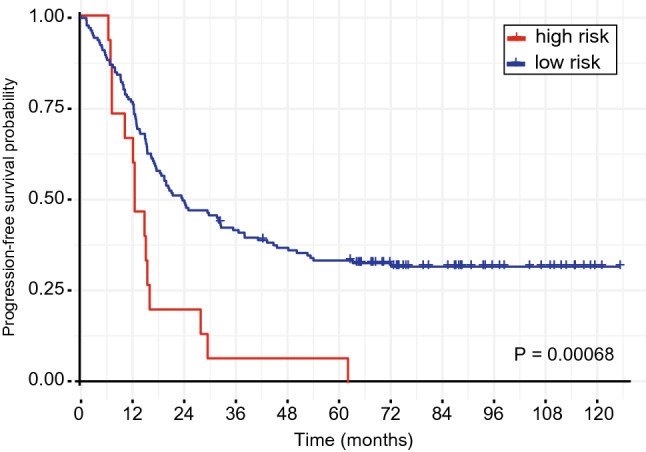
Progression-free survival for patients with overexpressed mRNA candidates. Survival curve shows that “high risk” (all overexpressed candidates) group have an overall shorter time to recurrence outcome compared to “low risk” (at least one candidate downregulated) in OC patients. P value is presented above.

Patients with short survival following therapy tend to present a more aggressive manifestation of the disease. To that end, we further evaluated progression-free survival (PFS) of patients by investigating whether the identified predictors would also be associated with time to relapse. Similar to OS, patients presenting overexpression of those markers also showed a shorter PFS compared to the remainder patients, with 60.0% (9/15) and 24.2% (36/149) relapse of the disease within the first 12 months, respectively (P = 0.0007) (Fig. 3). Moreover, we also investigated whether the overrepresentation of serous adenocarcinoma samples skewed our analysis. Similar to the previous results, no differences were observed between serous and non-serous histologic subtypes, potentially indicating a similar prognostic performance in the latter group (Supplementary Fig. S6).

Overall, our findings indicate that these 4 markers performed well in predicting patient OS and PFS in patients with epithelial OC. Noteworthy, given the relatively low representation of non-serous EOC subtypes in our cohort, these findings were not powered for discovery of histologic sub-type. Hence, it may only indicate the prognostic value in non-serous subtypes as well, needing further investigation.

### The candidate targets associate with poor prognostic features

In order to provide a better clinical significance, we next investigated whether the combination of *HSPA1A*, *CD99*, *RAB3A*, and *POM121L9P* would also be able to predict clinicopathological features mostly associated with poor patient prognosis. To that end, we evaluated the correlation between those 4 targets and tumour type (I and II), disease progression (whether disease progressed until last follow up), and primary platinum-based chemotherapy response (tumour recurrence within the first 6 months post-treatment and later) separately. We found that the combination of at least 2 of the selected targets were correlated with each of the clinical characteristics. The overexpression of *HSPA1A*, *CD99* and *RAB3A* showed to be associated with advanced tumour stage, tumour type II, tumour progression, and primary chemotherapy resistance (Table 3). None of the 4 predictors were shown to be associated with menopause status.

**Table 3:** Association of candidates with clinicopathological characteristics.

	*HSPA1A*		*CD99*		*RAB3A*		*POM121L9P*	
	OR	95% CI	OR	95% CI	OR	95% CI	OR	95% CI
Tumour stage	1.78	1.10–2.99	8.66	0.51–1.43	3.63	1.56–9.15	0.85	0.50–1.45
Tumour grade	3.84	2.03–7.9	5.68	0.30–1.02	3.93	1.44–11.76	0.80	0.42–1.57
Disease progression	2.12	1.28–3.65	1.52	0.89–2.70	4.04	1.66–10.59	1.14	0.64–2.08
Chemotherapy resistance	1.20	0.71–2.07	4.78	2.23–11.22	4.61	1.87–12.23	1.22	0.69–2.23
Menopause	1.03	0.62–1.70	0.98	0.57–1.76	0.61	0.24–1.45	0.81	0.43–1.41

*OR *odds ratio, *CI* confidence interval.

In order to investigate whether those 4 candidate markers would perform in a similar manner on other samples, we sought to validate our findings by two distinct strategies: (1) assessing them on an external cohort, and (2) comparing their performance with a second score index. On the first approach, we used a dataset from NCBI’s GEO database, namely GSE26193, comprised of 107 OC samples^21,22^. In line with our data, patients classified as “high risk” group did show a poorer overall survival when compared to the “low risk” group in this cohort, with 5-year OS of 0% and 34.5%, respectively (P < 0.0001) (Supplementary Fig. S7A). We further assessed the panel efficiency to discriminate between patients with OS longer or shorter than 5 years. In this cohort, the panel of markers showed a predictive performance of AUC = 0.67 (Supplementary Fig. S7B). For the second approach, we compared our classification with the 97 gene signature predicting chemotherapy response described by Matondo and colleagues^19^. Interestingly, by using their signature, the classification of “high-” and “low risk” groups were also observed in our cohort (Supplementary Fig. S7C). Furthermore, in agreement with our classification, the “high risk” group also presented a shorter OS than the “low risk” group (P = 0.001), with median values of 26.6 and 55.4 months, respectively, in comparison with 19.7 and 52.0 months found using our 4-target signature (Supplementary Fig. S7D). Finally, we assessed the gene signature performance from Matondo and colleagues with the validation cohort employed in our study, GSE26193. Similarly, we observed a distinct gene signature between the “high-” and “low risk” groups. The difference in OS was also observed between the two groups, although with a shorter interval of median OS between the groups, 29.1 months for “high risk” and 45.8 months for “low risk” (P = 0.044), contrasting with 13.0 and 41.9 months in the 4-target signature, respectively (Supplementary Figs. S7D, S8).

## Discussion

Given the current scenario of OC, of which approximately 65% of all cases are diagnosed at an advanced stage^2^, new biomarkers could be beneficial in order to improve the prognosis of patients. Hence, discovery of novel molecular targets still presents an overdue clinical challenge with unmet needs, despite helping to guide treatment decisions. Within the last decades, mRNA assessment has been widely used in the identification and development of novel molecular biomarkers for the diagnosis and treatment of a number of cancer types^23^. They can offer early and more accurate prediction and prognosis of the disease and its progression, allowing for the identification of individuals at risk. Hence, the assessment of the whole mRNA profile of a patient further provides the opportunity to identify not only unique biomarkers, but also the association among them. Here, in a prospective cohort of patients with EOC we investigated the association of global mRNA expression profiles primarily with OS, and extended the analysis to clinicopathologic characteristics associated with poor prognostics.

In OC, studies have indicated that its overall mRNA makeup is rather complex. A study cohort of 489 patients of serous adenocarcinomas categorized those patients in at least 4 distinct subgroups, according to their mRNA profile expression^24^. In a similar manner, other studies were able to indicate the association of OC with tumour subtype, grade, therapy response and OS^9,10,12,15,25^. Noteworthy, such studies did not investigate multiple clinicopathological characteristics in association with mRNA expression. In the present study, we sought to identify a distinctive set of dysregulated genes across different subtypes of epithelial OC associated with OS of patients. We further evaluated whether that set of candidate targets would also be correlated with patient clinicopathological features.

We investigated the mRNA profile of 196 patients with EOC by microarray technology. For a better prediction performance, the primarily discovered targets were submitted to a penalty-based scoring followed by a tenfold cross validation, in order to remove those weakly associated with OS or other targets. That resulted in the discovery of 4 candidates: *HSPA1A*, *CD99*, *RAB3A*, and *POM121L9P*. Overexpression of all targets associated with shorter patient survival. Such predictors also performed well in distinguishing PFS among patients.

Due to the complexity of OC, the longevity of a patient is highly linked to various clinicopathological outcomes, such as tumour type, the relapse of the disease and sensitivity to chemotherapy^26–29^. Here, we found that, with the exception of *POM121L9P*, the combination of at least two markers was capable to clearly differentiate between (1) type I and II tumours; (2) progressive and stable disease; and (3) chemo-resistant and -sensitive patients.

Interestingly, the association of any of these 4 predictors has not been previously reported in prior prognostic panels for OC. Among those predictors, *HSPA1A* is better known to be associated with other types of cancers, with some indications for OC^30,31^. *HSPA1A* is a member of heat shock proteins (HSPs) family, which are highly conserved throughout vertebrates and known as stress-inducible molecules, found overexpressed in a range of cancer types^32,33^. These molecules function mainly as chaperones, modifying the structure and interaction of other proteins^32,34^. HSPs also regulates the cellular apoptotic pathway, and the immune response^35^. Studies have reported that HSP expression is maintained at high levels in cancer as a consequence of the stress generated by hypoxia, genomic instability stresses, and abnormal translation of oncoproteins^36–38^. Hence, these proteins are critically involved in cancer cell proliferation, metastasis, invasion, and angiogenesis^32,33,38^. Here, we found the overexpression of *HSPA1A* in more aggressive OC cases, associated to high grade serous tumours, shorter time to progression of the disease and shorter OS. In contrast, an earlier study has shown that positive expression of *HSPA* resulted in a better mortality risk compared with negative expression in stage II oral cancer patients^39^. The disparity with the present results may be due to the fact that in that study patients were primarily grouped according to tumour stages and only *HSPA1A* expression was evaluated, whereas here we performed an unsupervised analysis among all known genes to find association within themselves and patient OS and PFS. It is still elusive on how *HSPA1A* contributes to an aggressive form of tumour, however some studies argue that its high levels in the cell might protect cancer cells from apoptosis, thus promoting tumour cell proliferation and migration^40^. Among our candidates, we also found *CD99* to be associated with tumour type and chemotherapy resistance, in addition to OS and PFS. This gene is expressed ubiquitously in many human tissues, and found significantly overexpressed in immature thymocytes, Ewing sarcoma, and peripheral neuroectodermal tumour^41,42^. It has further been suggested as a potential biomarker for diagnosis of ovarian granulosa cell tumours^43,44^. In a small cohort of 14 steroid-associate OC patients, Jones et al. found CD99 proteins to be strongly present and diffused among hematopoietic cells in all subjects^44^. Such observations were later confirmed in another study, where approximately 70% of cases also presented high levels of *CD99* expression^45^. Interestingly, this pattern has been observed only on a small subset of OC, on ovarian steroid cell tumours. Its function in tumour development is still elusive, but it has recently stirred more interest due to its involvement with immune response, cell differentiation, apoptosis and migration/invasiveness metastatic tumour cells^46^. CD99 plays a direct role in the transport and expression of major histocompatibility complex (MHC)^47^, and control of MHC-associated gene expression on dendritic cells, thus guiding immune response^46,48^. In vitro studies have shown that *CD99* overexpression was linked to cell mobility, invasion and adhesiveness by potentially affecting the cytoskeleton dynamics. It has been demonstrated that suppressing *CD99* expression by interfering RNA in glioma tumour cell lines markedly reduced cell migration, further suggesting that CD99 may contribute to the infiltrative ability of tumour cells^49^. Our results showed *RAB3A* expression to be associated with all clinicopathological features examined. Its association with cancer has scarcely been reported, thus its function is still widely unknown. No association with OC has been demonstrated. However, similar to our results, Kim and colleagues have also observed that high expression of *RAB3A* was present in tumour tissue from patients with glioblastoma multiforme, with significant association with high tumour grade^50^. Moreover, they investigated the role of this gene in mice models and observed that *RAB3A* affects tumour initiation, transformation and drug-resistance primarily by inducing cell cycle progression through cyclin D1 stimulation^50^. Furthermore, *RAB3A* is a member of the Ras-associated binding (RAB) family, known to be associated with more aggressive and treatment-refractory tumours. For instance, high expression of *RAB25A* has been observed in more than 88% of OC patients. Intriguingly, most of those patients were not disease-free following debulking surgery or primary chemotherapy and presented very short survival compared to other patients^51^. They further observed that knock-down of *RAB25A* in mice decreased the activation of the PI3K pathway and restored the expression of proapoptotic genes, which contributes to treatment sensitivity, apart from inhibiting cell proliferation. In a similar manner, our results showed that the overexpression of *RAB3A* associated with shorter OS, PFS, and poorer prognosis, including tumour type and cisplatin-based therapy resistance. We have also found *POM121L9P* expression to be linked to OS and PFS. However, when seeking for further clinical relevance, we did not find association of this gene with any clinicopathological characteristics.

We further sought to evaluate the performance of these 4 targets by (1) investigating them and their association with OS on an independent cohort, and (2) comparing their efficiency with a second signature-based score index^19^. In the former assessment, the classification established by the 4 targets was also able to distinguish between the “high-” and “low risk” groups. In line with our findings, the “high risk” group was associated with shorter OS. In the latter assessment, the classification by employing an external index score, a 97-gene based signature by Matondo and collaborators showed an equivalent outcome, where the “high risk” group associated with a poorer OS of patients. Noteworthy, our classification was able to identify a smaller number of individuals in both cohorts (18/196 in our cohort and 6/107 in the validation cohort) when compared to classification defined by the 97-gene signature (42/196 in our cohort and 34/107 in the validation cohort). Taking into consideration the number of targets from the current study, from a clinical applicability standpoint the use of a prognostic panel with fewer targets might be more cost- and time-effective than larger panels, whilst preserving its sensitivity. Here, we demonstrated that *HSPA1A*, *CD99*, *RAB3A* and *POM121L9P* performed well in predicting OS and PFS. We showed that the combination of all those 4 predictors performs better than each candidate alone. With the exception of *POM121L9P*, that combination further associated with a more aggressive form of OC—shorter PFS, high grade tumours and chemotherapy resistance. Moreover, considering that single biomarkers can be sensitive in predicting tumours at the cost of very low specificity, a panel of markers can provide a more accurate outcome. To that extent, we sought into determining gene predictors that could associate with a shorter OS and further poor prognosis in patients with OC.

In summary, we found that *HSPA1A*, *CD99*, *RAB3A* and *POM121L9P* expression profile distinguished OC patients in regard to OS and PFS. Furthermore, the overexpression of all those candidates associated with a poorer prognostic outcome, resulting in shorter OS and PFS, apart from high grade tumours, faster progression of disease and chemoresistance. Overall, this indicates that a panel comprised of a few predictors that associates with a more aggressive form of OC might be clinically relevant, by presenting a better performance than a marker alone. However, the current findings will benefit from further validation on more independent cohorts on whether such markers outperform the current ones in the clinic. Given the relatively small sample size used in the current study, those further evaluations will be crucial in examining the predictive efficiency of these 4 targets. In line with that, the value of devising a potential prognostic signature should provide accurate information about the patient, in order to aide in a more tailored treatment, and identify pathways of importance which can be effectively targeted for that therapy; these accomplishments in ovarian cancer are still elusive^13^. In that regard, the analysis of separate cohorts, or their combination in meta-analyses have shown encouraging alternatives^14^. Furthermore, it will be interesting to investigate whether those four reported markers present a similar performance in serum samples, potentially providing a better, mildly invasive approach to the patient. In this manner, it might show valuable for OC patient management and treatment.

## Methods

### Patients and samples collection

All tissue samples were obtained from patients enrolled in the Danish Pelvic Mass study, a national ongoing cohort initiated in 2004. Patients were diagnosed and surgically treated for epithelial OC between October 2004 and January 2010. Patients that had received primary cytoreductive surgery and where an epithelial histologic subtype had been confirmed were included in this study. The exclusion criteria were non-epithelial OC, neoadjuvant chemotherapy, scarce tissue material for analysis, and a history of another cancer type. All samples obtained were examined by a specialized pathologist, and patients were registered in the Danish Gynaecologic Cancer Database (DGCD), a national compulsory clinical database, as well as in Bio- and GenomeBank, Denmark (RBGB, www.regioner.dk), a registry including clinical biobanks, ensuring biological material of high quality for patients own treatment and biomarker research. The study was carried out according to the guidelines of the Declaration of Helsinki, including written informed consent from all participating patients, and it has been approved by the Danish National Committee for Research Ethics, Capital Region (approval codes KF01-227/03 and KF01-143/04). All patients were followed until either death of any cause, emigration or January 17th, 2015.

Progression Free Survival (PFS) was established as the time spanning from primary surgery until relapse, disease progression/progression of disease (PD) or death of any cause, based on the event which occurred first. The relapse and PD were characterized by performance of best clinical evaluation, assessed by CT/MRI/PET-CT scans, serum CA-125 and patients’ symptoms. In cases where second line chemotherapy was initiated, lacking relapse or PD information, that timepoint was considered as relapse or PD. Chemotherapy-resistance was defined by the event of relapse or PD within 6 months following chemotherapy. Conversely, chemotherapy-sensitive subjects were defined as either presenting no relapse or PD, or if occurred more than 6 months following the end of first line chemotherapy. The timespan from end of first line chemotherapy until relapse, PD or start of second line chemotherapy was designated time to progression. Cases with time to progression more or less than 6 months were classified in two different groups, further used for statistical analyses of resistance to chemotherapy. Cause of death was defined as either, death of gynecologic cancer, or death of other causes.

Tumour tissues were stored as formalin-fixed and paraffin-embedded (FFPE), and all samples registered in RBGB/Danish CancerBiobank. A specialized pathologist in gynaecology has revised histologic diagnoses for all tissue samples. By conventional haematoxylin and eosin staining, all samples included presented a tumour presence above 50%.

### RNA extraction and gene microarray profiling

Total RNA was extracted from 20 µm thick FFPE tumour sections using the RecoverAll Total Nuclei Acid Isolation Kit for FFPE samples (Ambion, USA). One sample was discarded, due to insufficient material amount, resulting in a total of 196 profiled cases. Samples were then hybridized to Affymetrix GeneChip Human Genome U133 Plus 2.0 Array (Affymetrix, USA), following the manufacturer instructions. Briefly, RNA samples were subjected to two cycles of cDNA conversion, amplified and labelled with biotinylated ribonucleotide analogues, generating cRNA single strands. Synthesised strands were then purified, heat-induced fragmented and finally hybridized to the microarray chip. Microarrays were scanned in a Genechip Scanner (Affymetrix, USA), and data acquisition was performed by GeneChip Command Console (Affymetrix, USA).

### Data treatment and statistical analysis

Raw data were background-corrected and normalized and log-transformed by applying the RMA method^52^, followed by data cleaning (removal of control probes), resulting in a total of 54,613 probes. Normalized data were primarily submitted to Cox univariate regression, identifying 2,837 probes (P < 0.01). Due to the large number of predictors, a LASSO-penalized model for Cox multivariate regression was applied, and the resulting targets were finally cross-validated (tenfold) by a last round of Cox multivariate analysis. The primary outcome for the investigation of candidate biomarkers was OS, defined as time in months, counting from the time of diagnosis to time to death, or last censored follow-up. The association between the candidate targets and the clinicopathological features of the patients were investigated by multivariate logistic regression model, considering all discovered targets combined for each feature alone. The clinical characteristics assessed were tumour type, progression of disease, resistance to first line of chemotherapy, and menopause status. All analyses with the clinical features were adjusted for age. Receiver operating characteristic (ROC) curves were calculated in order to assess the efficiency of prediction of our models presented by the area under the ROC (AUC). Targets with P < 0.01 were considered associated with patient survival, and a penalty alpha = 1.0 was applied for the LASSO method. For the classification of groups, the median expression value for each target was employed in order to minimize for the effect of possible expression outliers. All statistical analyses were performed in the R environment^53^.

### Independent cohort assessment

In order for validation with external data, OC samples from an independent cohort were used in order to assess transcription expression and OS association of the candidate markers. The cohort was comprised by 107 OC tissue samples^21,22^. The data was retrieved from the Gene Expression Omnibus (GEO) database, with their corresponding identification GSE26193. Moreover, for comparison of performance with other index scores, we used a 97-gene expression signature from Matondo and collaborators^19^. The normalized matrix data were directly retrieved from the GEO database for further use in the analyses.

## Supplementary Information

Supplementary Information
