# Supplementary material for: Gene expression profile association with poor prognosis in epithelial ovarian cancer patients
Source: Sci Rep. 2021 Mar 8;11:5438. doi: 10.1038/s41598-021-84953-9 (PMC7940404; doi:10.1038/s41598-021-84953-9)
Supplement: Supplementary file 1 — Supplementary Information [file 41598_2021_84953_MOESM1_ESM.pdf]

Gene expression profile association with poor prognosis in epithelial ovarian cancer patients

Douglas V.N.P. Oliveira [1], Kira P. Prahm [1], Ib J. Christensen[1], Anker Hansen[3], Claus K. Høgdall [2], Estrid V. Høgdall [1,*]

[1] Department of Pathology, Herlev Hospital, University of Copenhagen, Herlev, Denmark

[2] Department of Gynaecology, Juliane Marie Centre, Rigshospitalet, University of Copenhagen, Copenhagen, Denmark

[3] Oncology Venture, Denmark

* corresponding author: estrid.hoegdall@regionh.dk

Key words: global mRNA profiling, biomarker, ovarian cancer.

Running title: Global mRNA profile in epithelial ovarian cancer.

Supplementary information

Supplementary Table S1

Supplementary Figure S1

Supplementary Figure S2

Supplementary Figure S3

Supplementary Figure S4

Supplementary Figure S5

Supplementary Figure S6

Supplementary Figure S7

Supplementary Figure S8

Histologic subtype	HSPA1A		CD99		RAB3A		POM121L9P	
	Median	Std.Dev.	Median	Std.Dev.	Median	Std.Dev.	Median	Std.Dev.
Clear Cell	9.456	0.812	9.348	0.611	5.789	0.292	3.364	0.437
Endometrioid	9.121	0.576	9.248	1.049	5.718	0.420	3.153	0.461
Mucinous	9.668	0.629	9.188	0.268	5.781	0.431	3.498	0.389
Serous	9.933	0.724	8.949	0.623	5.929	0.427	3.284	0.714

Supplementary Table S1. Median expression values of *HSPA1A*, *CD99*, *RAB3A* and *POM121L9P* in each histologic subtype. Std.Dev.= standard deviation.

Supplementary Figure S1

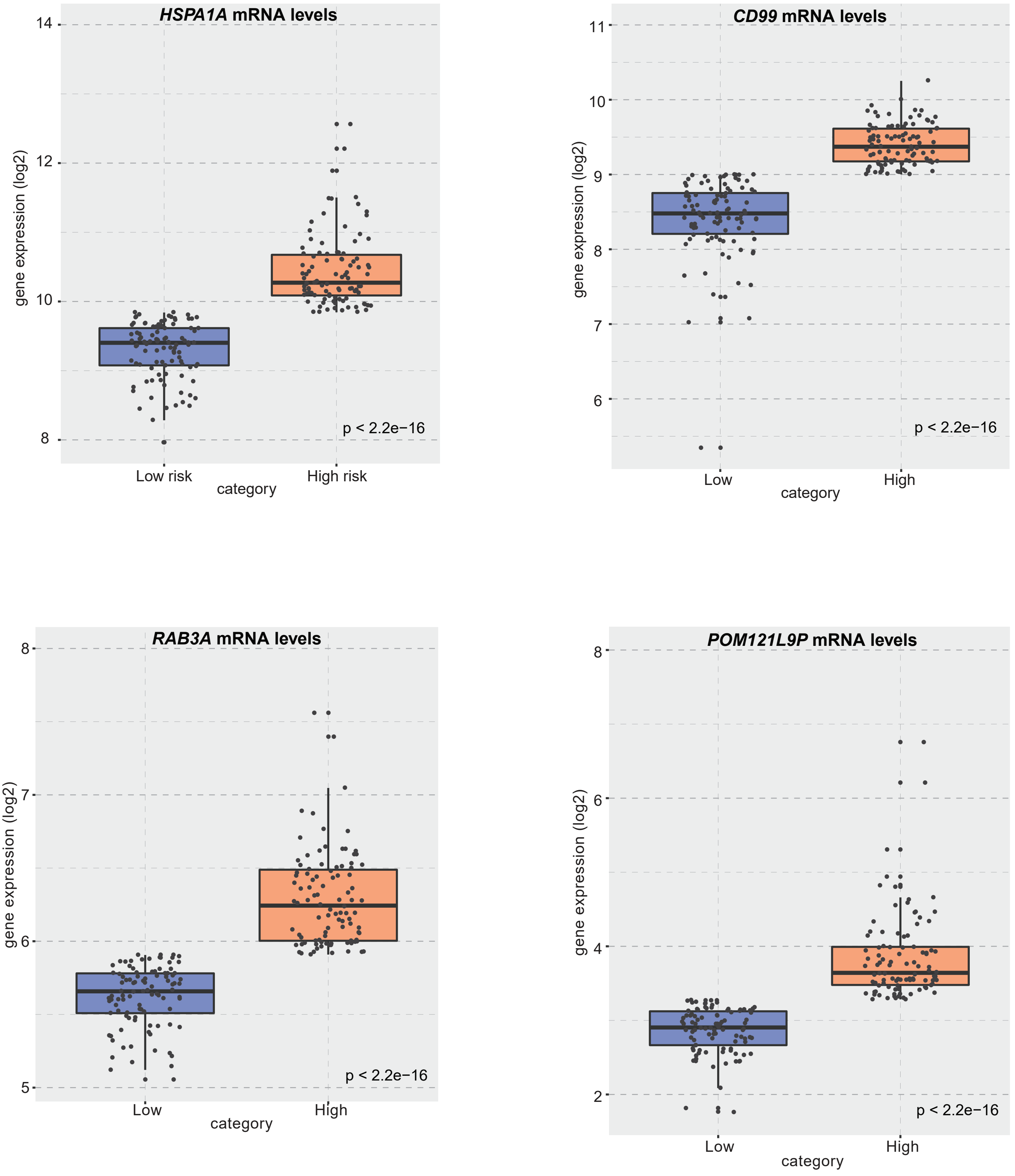

Supplementary Figure S1. Gene expression in “low risk” (blue) and “high risk” (orange) groups. Values are normalized and provided as log2 and P-value is presented above.

Supplementary Figure S2

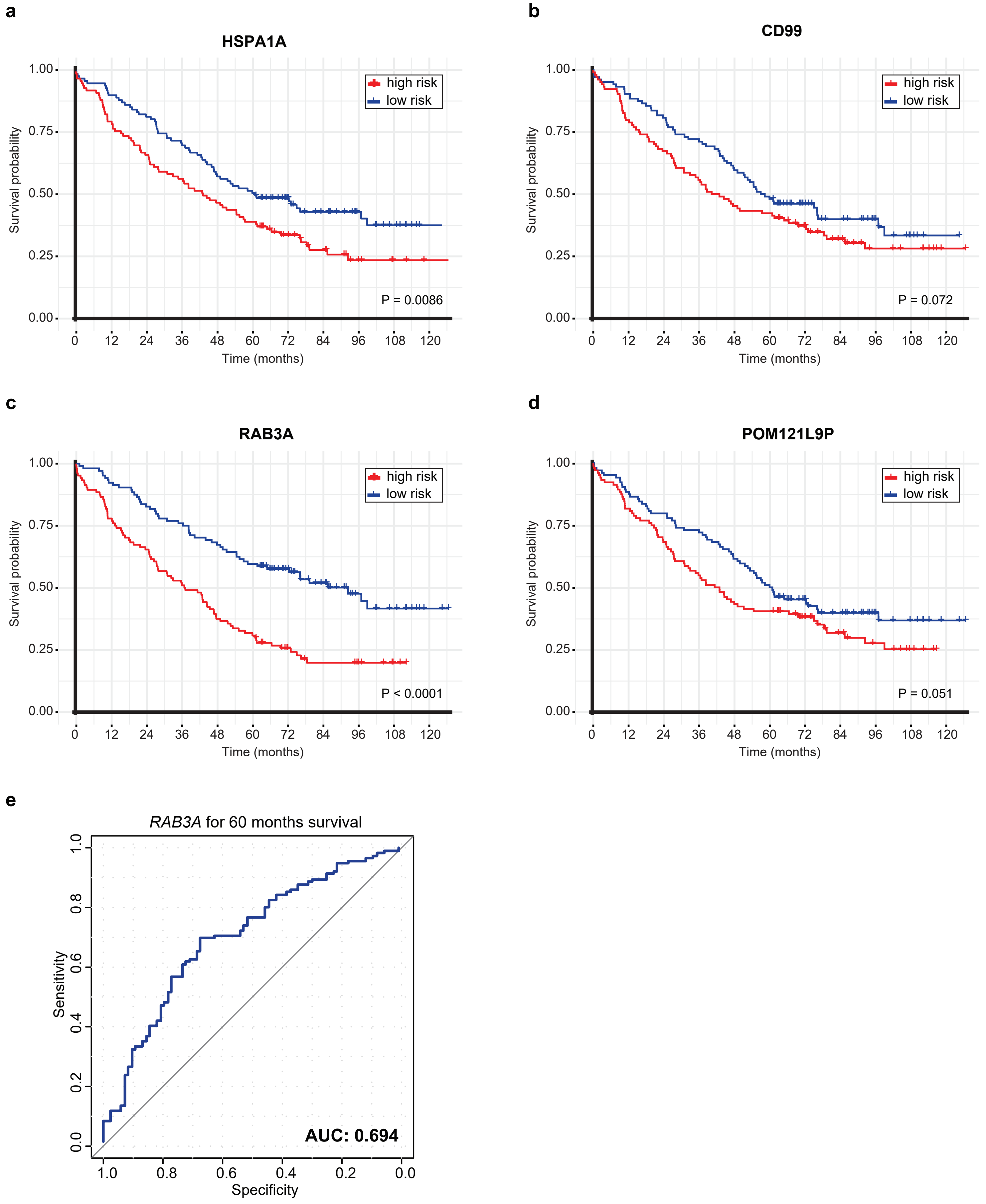

Supplementary Figure S2. Overal survival curves for each candidate (*HSPA1A*, *CD99, RAB3A* and *POM121L9P*) individually in "high risk" (red) and "low risk" (blue) groups (a-d). (e)ROC/AUC for RAB3A alone for 60 months patient OS. P-values are presented above.

Supplementary Figure S3

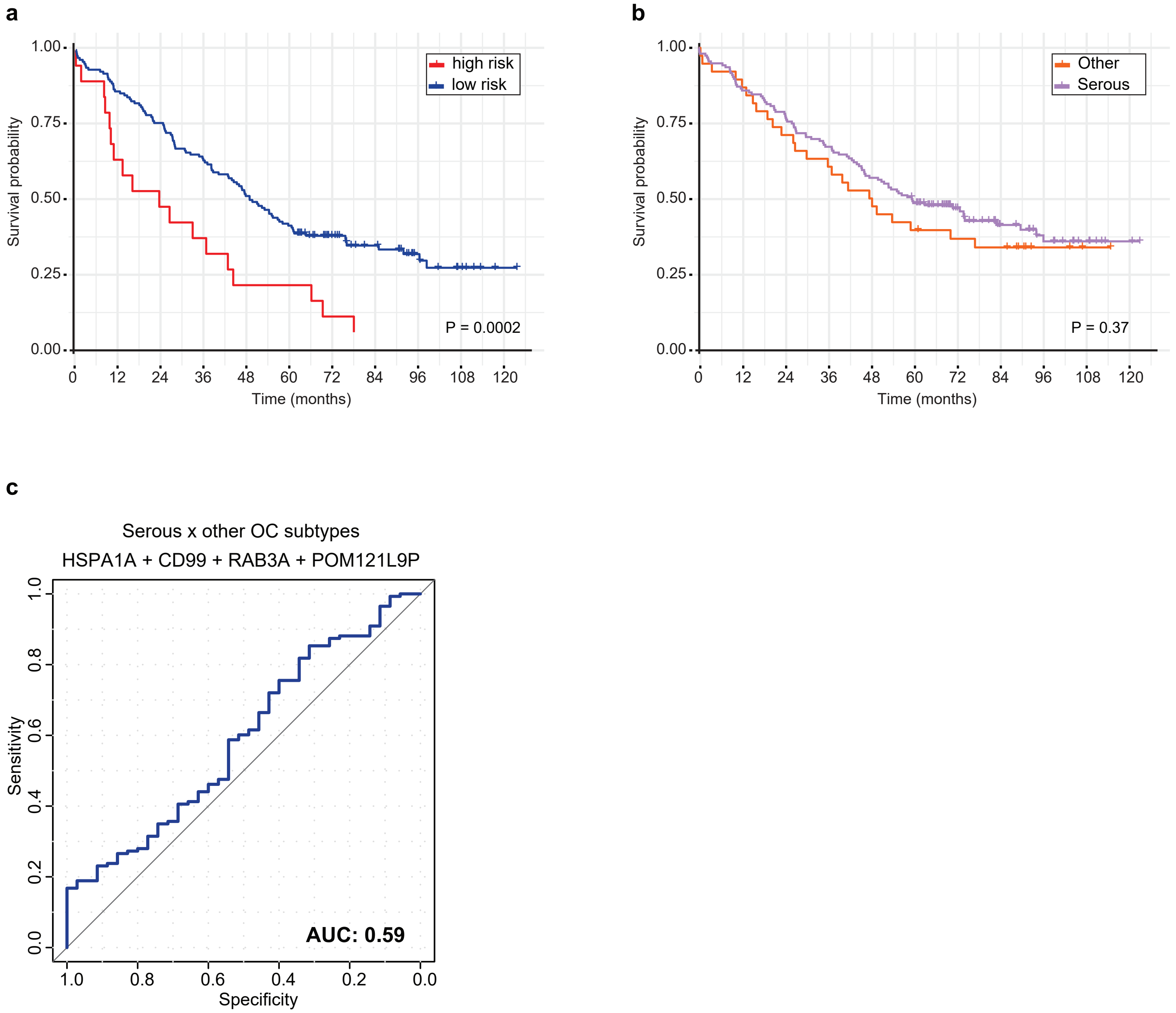

Supplementary Figure S3. (a) Survival curve between “high risk” (red) and “low risk” (blue) in serous adenocarcinoma subtype. (b) Survival curve between serous adenocarcinoma and other OC subtypes. (c) AUC/ROC curve of combination of HSPA1A, CD99, RAB3A and POM121L9P in serous adenocarcinoma x other OC subtypes. P-values and AUC are presented above.

Supplementary Figure S4

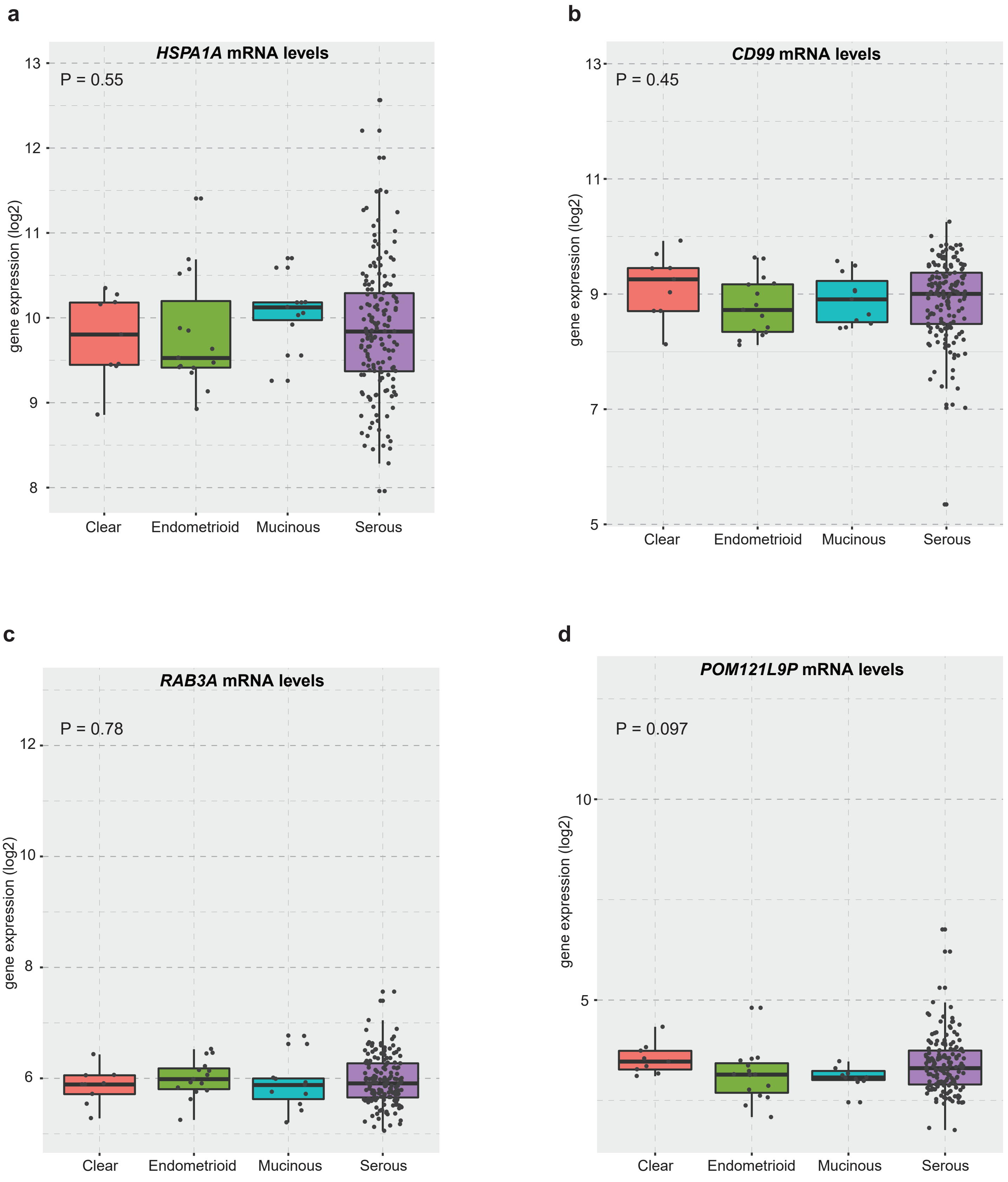

Supplementary Figure S4. Gene expression in all OC subtypes for *HSPA1A* (a), *CD99* (b), *RAB3A* (c) and *POM121L9P* (d). Values are normalized and provided as log2 and P-values are presented above.

Supplementary Figure S5

a

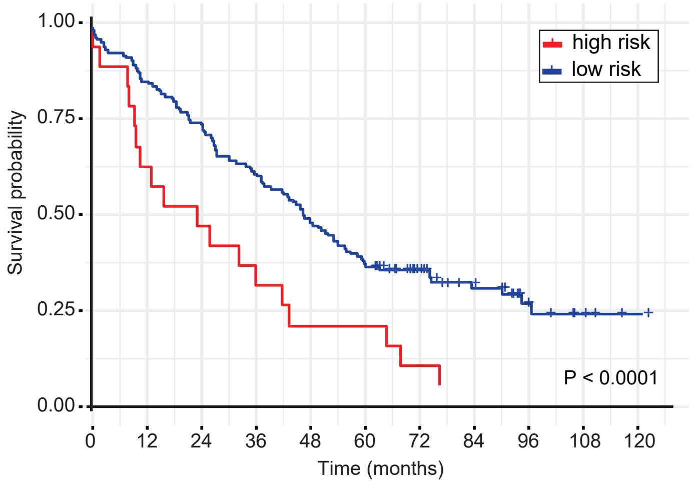

Supplementary Figure S5. Survival curve between “high risk” (red) and “low risk” (blue) in high grade serous adenocarcinoma subtype. P-value is presented above.

Supplementary Figure S6

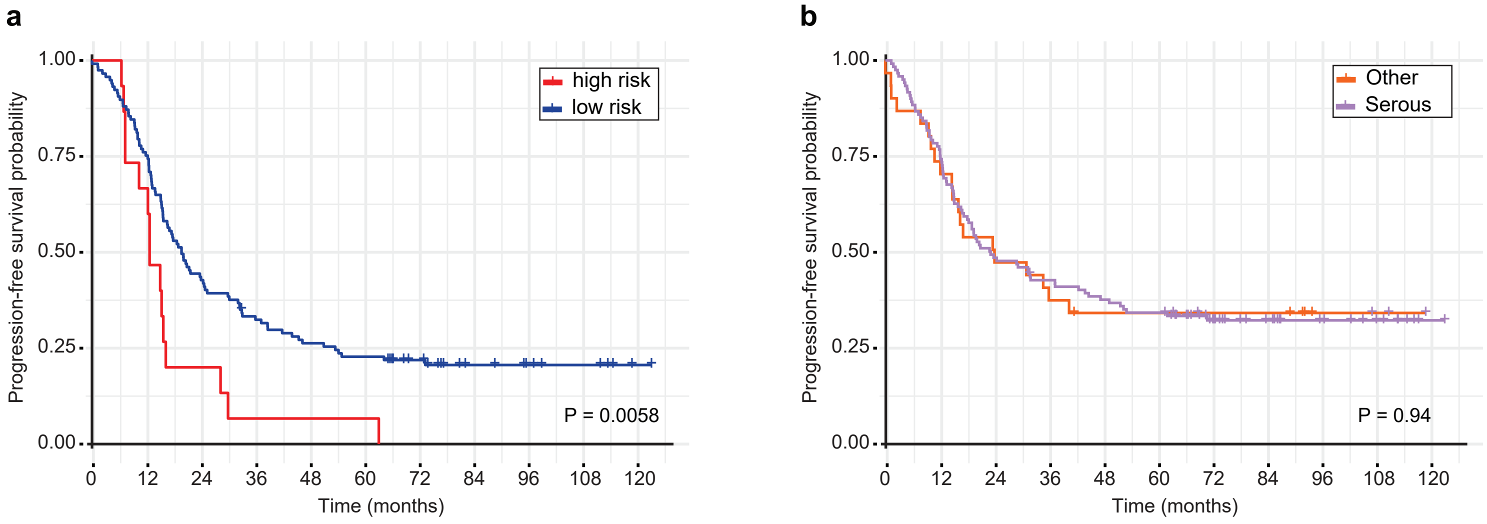

Supplementary Figure S6. (a) Progression-free survival curve between “high risk” (red) and “low risk” (blue) in serous adenocarcinoma subtype. (b) Progression-free survival curve between serous adenocarcinoma and other OC subtypes. P-values are presented above.

Supplementary Figure S7

a

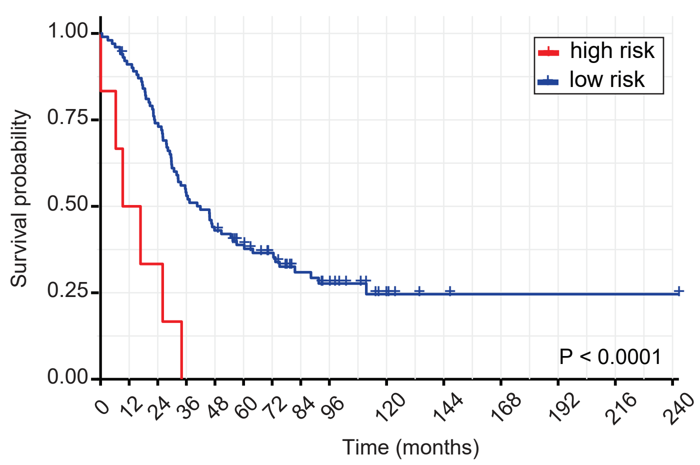

b

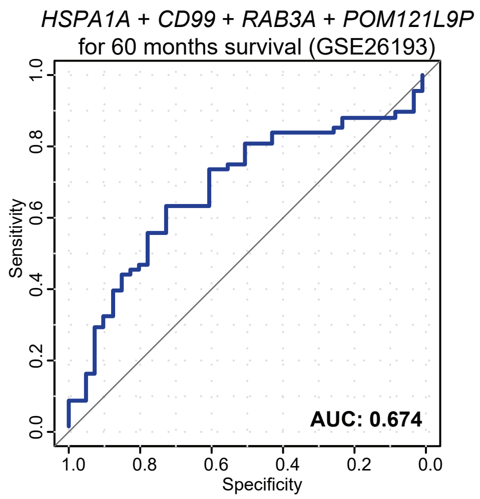

c

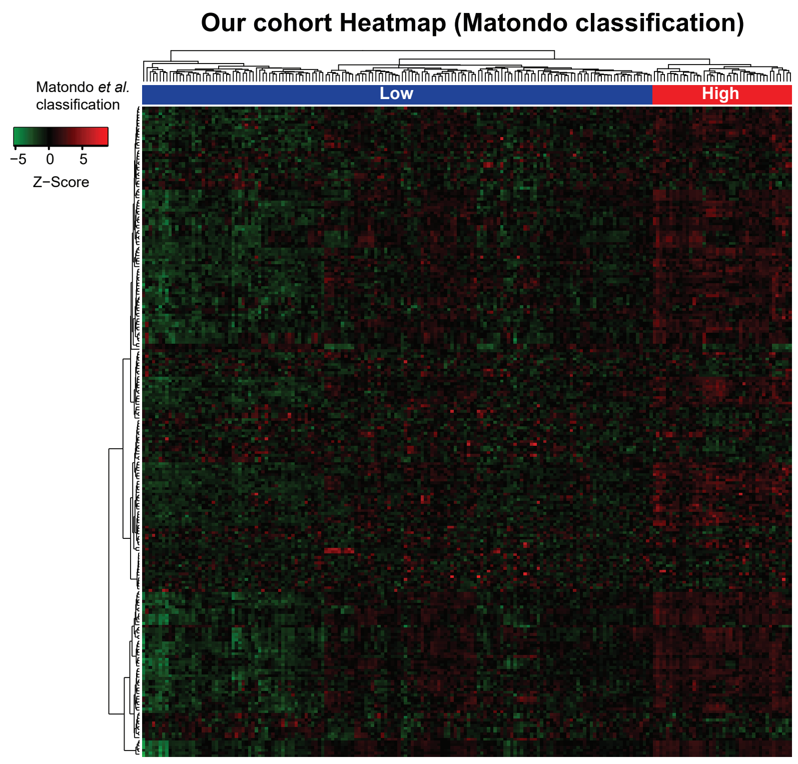

d

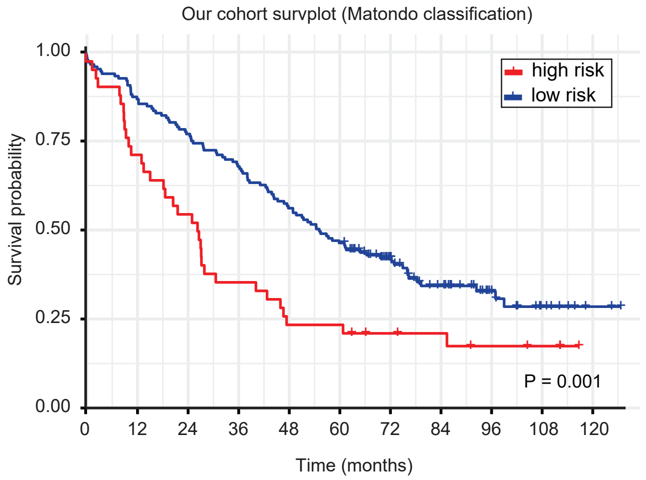

		196 patients cohort			GSE26193 cohort		
	Classification	median	lower	upper	median	lower	upper
4 targets signature	High_risk	19.74	9.86	44.38	13.03	6.37	
	Low_risk	51.98	44.52	64.93	41.89	32.49	57.10
Matondo *et al*. signature	High_risk	26.60	18.38	42.87	29.08	20.73	50.69
	Low_risk	55.38	47.61	72.93	45.77	33.68	75.10

Supplementary Figure S7. Validation study. (a) Survival curve shows that “high risk” (all candidates overexpressed) group has a shorter survival compared to “low risk” OC patients, and (b) AUC/ROC curve of combination of HSPA1A, CD99, RAB3A and POM121L9P for 5-year OS in cohort GSE26193 (n= 107). The 97-gene signature performance from Matondo et al. (2017) in our current cohort (n=196) showing (c) Heatmap and classification of "high-" (red) and "low risk" (high) groups. (d) K-M curve for the "high-" and "low risk" groups, and table with median OS of patients on both cohorts and both signature panels. P-values and AUC are presented above.

Supplementary Figure S8

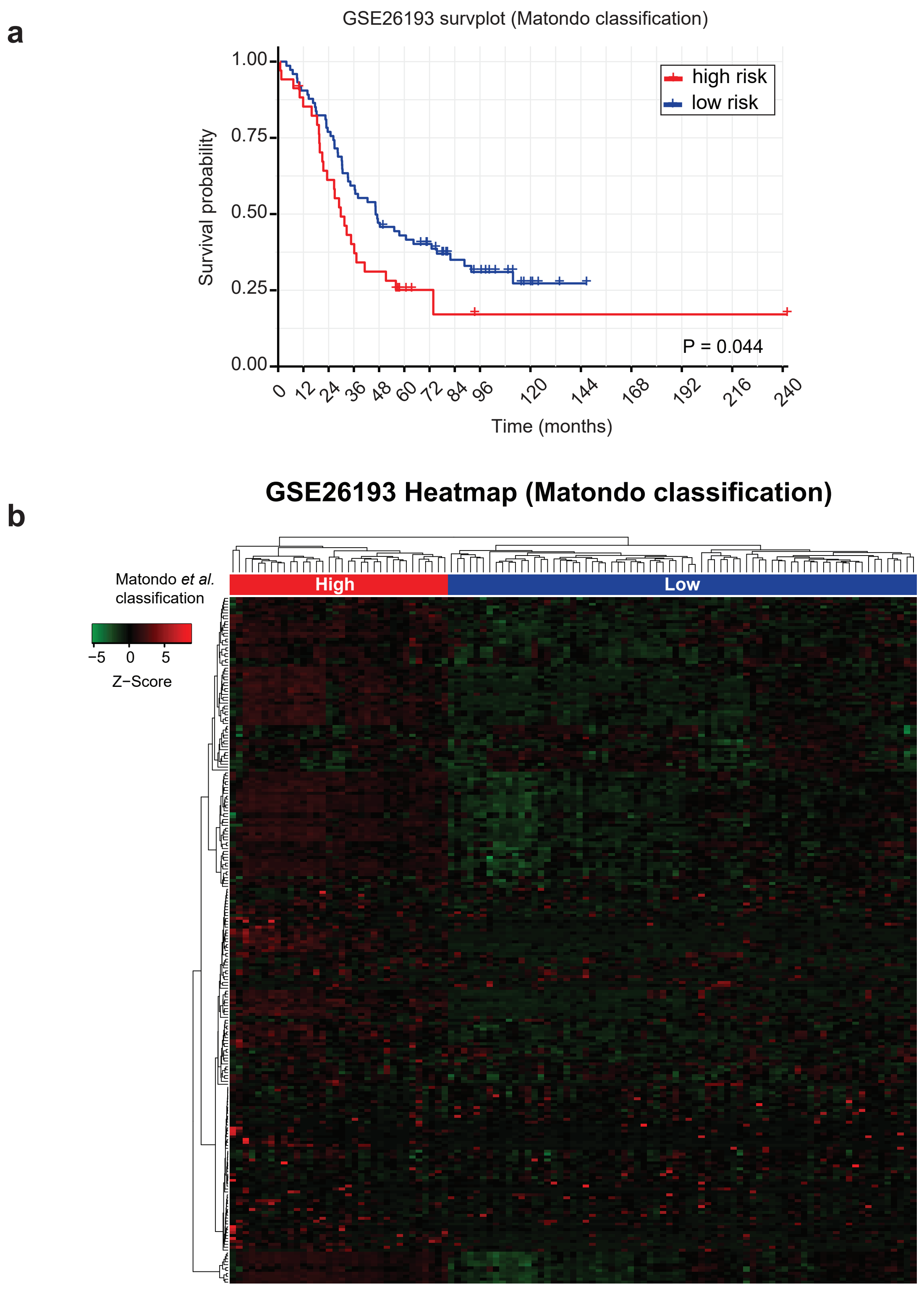

Supplementary Figure S8. Assessment of the 97-gene signature panel from Matondo and colleagues on cohort GSE26193 (n= 107). (a) Survival curve shows that “high risk” group has a shorter survival compared to “low risk” OC patients. (b) Heatmap and classification of "high-" (red) and "low risk" (high) groups. P-value is presented above.
